# Elucidating endotoxin-biomolecule interactions with FRET: extending the frontiers of their supramolecular complexation

**DOI:** 10.14440/jbm.2017.172

**Published:** 2017-04-28

**Authors:** Eugene M. Obeng, Elvina C. Dullah, Nur Syahadatain Abdul Razak, Michael K. Danquah, Cahyo Budiman, Clarence M. Ongkudon

**Affiliations:** 1 Biotechnology Research Institute, University Malaysia Sabah, Kota Kinabalu, Sabah 88400, Malaysia; 2 Department of Chemical Engineering, Curtin University Sarawak, Miri, Sarawak 98009, Malaysia

**Keywords:** biomolecular interaction, endotoxicity, endotoxin, FRET, lipopolysaccharide

## Abstract

Endotoxin has been one of the topical chemical contaminants of major concern to researchers, especially in the field of bioprocessing. This major concern of researchers stems from the fact that the presence of Gram-negative bacterial endotoxin in intracellular products is unavoidable and requires complex downstream purification steps. For instance, endotoxin interacts with recombinant proteins, peptides, antibodies and aptamers and these interactions have formed the foundation for most biosensors for endotoxin detection. It has become imperative for researchers to engineer reliable means/techniques to detect, separate and remove endotoxin, without compromising the quality and quantity of the end-product. However, the underlying mechanism involved during endotoxin-biomolecule interaction is still a gray area. The use of quantitative molecular microscopy that provides high resolution of biomolecules is highly promising, hence, may lead to the development of improved endotoxin detection strategies in biomolecule preparation. Förster resonance energy transfer (FRET) spectroscopy is one of the emerging most powerful tools compatible with most super-resolution techniques for the analysis of molecular interactions. However, the scope of FRET has not been well-exploited in the analysis of endotoxin-biomolecule interaction. This article reviews endotoxin, its pathophysiological consequences and the interaction with biomolecules. Herein, we outline the common potential ways of using FRET to extend the current understanding of endotoxin-biomolecule interaction with the inference that a detailed understanding of the interaction is a prerequisite for the design of strategies for endotoxin identification and removal from protein milieus.

## INTRODUCTION

Cell culture contamination has been a prevalent issue in most bioproduct manufacturing processes, especially in companies producing cell culture-based product such as vaccines and injectable drugs. One of the topical chemical contaminants of major concern to researchers, especially in applications involving Gram-negative bacteria bioprocessing, is endotoxin. In biotechnology, endotoxin detection and removal from recombinant proteins are critical and challenging steps in the preparation of bioproducts such as vaccines, injectable therapeutics, and useful food supplements. The reason is that endotoxin is a natural component of the bacterial expression systems widely used in manufacturing therapeutic proteins [1]. However, the presence of endotoxin in intracellular products is a liable cause of pyrogenic discomforts to mammalian bodies ranging from fever to irreversible and fatal septic shocks. The endotoxin pathophysiological consequences have necessitated the necessary monitoring and control of endotoxin levels to get rid of the associated dangers in all stages of bioprocessing [2,3]. Simply put, the presence of endotoxin in media interacts with various target proteins based on charge and or affinity differences (*e.g.*, hydrophilicity, hydrophobicity, amphipathic attributes, *etc*.) and consequently cause detrimental end-results. In the production of recombinant biomolecules (using Gram-negative bacteria) for pharmaceutical and biomedical purposes, a significant level endotoxin remains with the bioproduct, even after purification. Sometimes, further stringent purification results in a compromise on product quality and quantity. This brings to fore the belief that there may be some localization and time-dependent interactions holding lipopolysaccharide (LPS) and these molecules together in forms that make them difficult to separate. Some of the relevant sectors facing endotoxin associated challenges are commonly the pharmaceutical and therapeutic products manufacturing industries, the food industries, and the potable water manufacturing industries. By virtue of the dangers, it has become imperative for researchers to engineer reliable means/techniques to detect, separate and remove undesirable traces of endotoxin from aqueous solutions, without compromising the quality and quantity of the end-product.

Several attempts such as the study of endotoxin-biomolecule interactions [4,5] and endotoxin-biomolecule separation techniques [6-8] are still being pursued in the bid to solve the threats posed by endotoxin. However, the basis for the development of the latter is comprehensively dependent on a thorough understanding of the former: the interplay between the endotoxin and the biomolecule. The understanding of the interaction between endotoxin and biomolecules still suffers a great dearth. As a result, the existing endotoxin identification, quantification, and subsequent removal techniques also suffer considerable limitations (refer to **Table 1**). However, Förster resonance energy transfer (FRET) has the potential to bridge the gap, owing to its notable achievements in protein and other biomolecular studies. Herein, the article introduces the structure of endotoxin and some associated mechanistic consequences and accentuates the prospects of FRET as a promising complementary technique for the identification and analysis of endotoxin-biomolecule interaction. Also, the article presents some of the limitations of the existing detection methods for endotoxin in protein milieu and suggests how FRET could help improve the drawbacks. In no way does this article claim itself as a complete review of endotoxin and FRET; however, the essential concepts necessary for prefacing the main idea—the need to extend the frontiers of endotoxin-biomolecule interactions with FRET—have been addressed. Due to space constraints, the reader’s attention would be drawn to relevant literature with regards to principles, protocols, and technical details.

## ENDOTOXIN AND ITS COMPOSITION

Endotoxin is a saccharolipid which constitutes the most abundant component of the outer membrane of most Gram-negative bacteria [9,10]. It forms an inherent part of the bacterial outer membrane, and it is responsible for the stability and organization of the microbe [11]. The presence of endotoxin in the outer cell envelope is necessary for bacterial growth and viability [12,13]. Functionally, endotoxin contains the main virulent factor of the bacteria and serves as a protective barrier which shields the bacteria from dangerous chemicals such as antibiotics [12,14-17].

**Figure 1.**
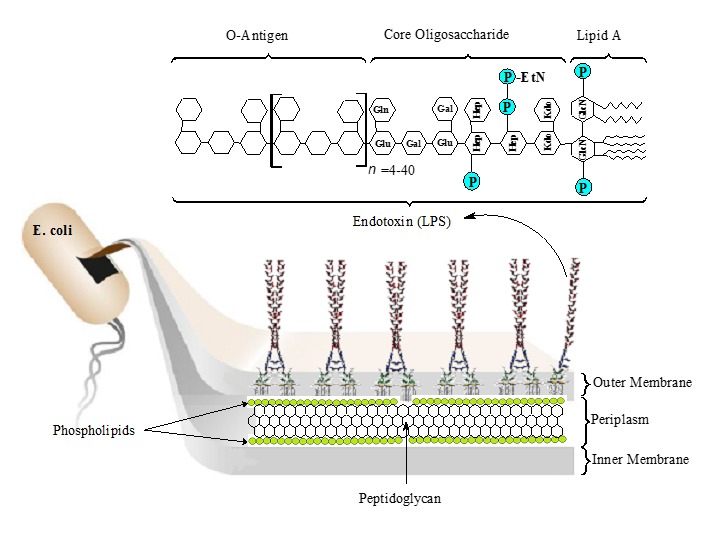
A typical structure of LPS. The cell envelope of Gram-negative bacteria consists of outer and inner membranes which sandwich layers of peptidoglycan within a space, called periplasm. The outer membrane is asymmetrically bilayered, consisting of phospholipids in the inner leaflet and mostly LPS in the outer leaflet. The LPS consists of lipid A moiety connected by ester amine linkage (GlcN) to a polysaccharide moiety. Current structural chemistry of LPS has shown clear differences in the compositional makeup of LPS. The reader is referred to Klein *et al*. [27,37] for the common structural forms. Kdo: 3-deoxy-D-manno-2-octulosonic acid; Hep: L-glycero- D-manno-heptose; P: phosphate; P-EtN: phosphoethanolamine; Glu: D-glucose; Gal: D-Galactose.

Suppressor-free Endotoxin is most often referred to as LPS (**Fig. 1**). Both names, endotoxin and LPS, are considered synonymous even though LPS is only the component in the endotoxin that stimulates the toxicity effect [18]. Mostly, LPS is amphiphilic in nature and consists of a lipid A fraction that is hydrophobic and a polysaccharide segment that is hydrophilic [19-21]. These hydrophobic and hydrophilic characteristics are interestingly two contrasting physicochemical properties. Notably, the LPS structure is highly heterogeneous, but there is a common architectural similarity in the toxicity factor, called lipid A. The lipid A moiety is a glucosamine-based phospholipid which is anchored into the outer leaflet of the asymmetrically bilayered outer membrane [22]. Lipid A is well-known to be responsible for the bioactivity of endotoxin [22-24]. In pure preparations, the lipid A portion assumes a systemized hexagonal arrangement, and it is more structurally conserved compared to the polysaccharide chain [8,13]. Nevertheless, Wong *et al*. (1979) [25] has reported unusual discrepancies in the structure and characteristics of the lipid A of the Legionnaires’ disease (LD) bacterium, which happen to be inconsistent with that of the classical lipid A of most Gram-negative bacteria. The LD bacterium was reported to have branched-chain fatty acids associated with its lipid A instead of the peculiar hydroxy fatty acids; therefore, making the associated toxicity principle a putatively new type of bacterial LPS. According to Martirosyan *et al*. [26], LPS with hexa-acyl lipid A moiety is more ‘endotoxic’ than tetra-acyl LPS. Although there are reported occurrences of non-stoichiometric (covalent) modifications (*e.g.*, the inclusion of phosphoethanolamine (P-EtN) and 4-amino-4-deoxy- L-arabinose (L-Ara4N)), the toxicity principle of endotoxin as well as the enzymes assisting its biosynthesis exhibits the greatest structural conservatism compared with that of the core sugars and the O-antigen [13,27,28].

The hydrophilic polysaccharide moiety consists of a non-repeating core oligosaccharide and a repeating polysaccharide, called O-antigen. The polysaccharide moiety helps the bacteria to resist antimicrobials and other environmental stresses [22]. For most species, especially the wild-type strains, the LPS structure is most often composed of lipid A, core oligosaccharide, and O-specific polysaccharide chain covalently linked together [12]. Any strain with all the three distinct regions in place is designated as ‘smooth’ whereas ‘rough’ is the term for the strains devoid of O-antigen (*e.g.*, *E. coli* K-12). However, strains without lipid A are not known [8,29-31]. According to Petsch and Anspach [7], the structural differences do not blight the viability of the bacteria likewise the biochemical effectiveness of endotoxin. However, severe defects in LPS biogenesis induce envelope stress response [32].

For the core oligosaccharides, they are sequentially assembled at the cytoplasmic surface of the inner membrane on lipid A in a process that involves some membrane-associated glycosyltransferases to function as coordinated complexes using nucleotide sugars as donors [23,33]. The core oligosaccharides have a rigid structure with an inner 3-deoxy-D-manno-2-octulosonic acid (Kdo) cum L-glycero-D-manno-heptose (Hep) region and an outer hexose assembly that is more structurally multifarious than those of the inner core [8,33]. In consonance with lipid A, non-stoichiometric alterations in the inner core oligosaccharide are often observed. For instance, Frirdich *et al*. [34] and Müller-Loennies *et al*. [35] have reported truncations in the outer core as a result of the (2→4)α-Kdo polysaccharide assuming a trimeric form instead of the common dimer forms. The presence of glutamine (Gln) metabolites in the core oligosaccharide is conceivable. This supposition stems from the fact that glutamine metabolites are of central importance for normal cellular functions such as cell protection, cell repairs, and cell growth [36]. The core oligosaccharide portion close to lipid A and lipid A itself are partially phosphorylated and, thus, conferring a residual negative charge to the endotoxin [8,37]. This anionic charge stemming from the inherent phosphate groups makes it possible, even in theory, for endotoxins to be removed by a counter-ionic approach. As an exception, the first LPS with an overall zero net charge has been discovered [38]. This resulted from an observed counter balance between ethanolamine substituents (+) and phosphate residues (**−**) [38].

The common nature of Kdo within the core oligosaccharide and its indispensable role in conserving the integrity and viability of the bacterial outer membrane have led to the extensive investigation into its synthesis [12,14,15,39]. For instance, it has been discussed that dissimilarities in the structure of the core oligosaccharide of different strains and genera of bacteria contribute to the permeability barrier of the cell and, thus, indicating the protective role of the polysaccharide chain [32]. In other words, the physico-chemical behavior of the entire structure of LPS is guided by the absence, presence, and alteration in the polysaccharides (including Kdo) as evinced in [14,15].

Similar to the core oligosaccharides, the O-specific antigen is synthesized at the cytoplasmic surface of the inner membrane. Glycosyltransferases assemble O-antigen units on the membrane-bound carrier (*i.e.*, undecaprenyl phosphate) which is also used for the synthesis of capsular polysaccharides and peptidoglycan [33]. The O-antigen of LPS exhibits significant diversity; therefore, the units can be homopolymers or heteropolymers [33]. Moreover, there are also noticeable differences in the position and stereochemistry of the O-glycosidic linkages and, therefore, the connection of units may be linear or branched [17].

It is worth emphasizing that there is considerable heterogeneity in endotoxin, even when isolated from the same bacteria culture, genus or species [40,41]. This may be attributed to environmental stresses which influence the length and composition of mostly the hydrophilic constituents. The diverse chemical and structural forms of LPS (*i.e.*, glycoforms I, IV, and V) and associated elucidations have been reported in the literature, and the various genes and their products have been identified to confer different microbial characteristics [27,37]. Owing to the variable length of the polysaccharide chain, the molecular weight of an endotoxin unit may vary from 10 to 20 kDa; even extreme masses of 2.5 kDa (for O-antigen-deficient) and 70 kDa (for very long O-antigen) are possible [8].

## PATHOPHYSIOLOGICAL CONSEQUENCES

The endotoxin pathophysiological effects, termed endotoxicity, are the associated pyrogenic discomforts such as septic shock, tissue injury and even death of host organism. The pathology of the discomforts (*i.e.*, severe sepsis and septic shock) is the repercussion of an unbalanced response of the host to pathogen-associated molecular patterns (PAMPs) [42]. These PAMPs may be of immunological significance if the endotoxin concentration in the mammalian cell is relatively low. However, high endotoxin concentrations trigger multiple events that result in multi-organ failure and septic shock which are difficult to cure [3].

In a simplistic view, Rudyk *et al*. [43] asserted that the cardinal feature of sepsis is an oxidative stress characterized by decreased peripheral vascular resistance, systemic inflammation, microvascular leakage, and reduced cardiac output, resulting in high blood vessel permeability and enlargement. Their work further explained that the combined effect of the systemic change is hypotension, which has a high possibility of culminating in death. Magi *et al*. [44] also established that persistent infections (*e.g.*, sepsis, endocarditis, and myocarditis) caused by bacterial stressors such as endotoxin stimulate the development of cardiac hypertrophy by altering intracellular Ca^2+^ homeostasis in the heart. The process directly involves Na^+^/Ca^2+^ exchanger1 (NCX1) within the heart. All these perturbations and discomforts associated with endotoxin justify the need to research and get rid of this ‘trouble-maker’ in all aspect of biomolecular processes.

**Figure 2.**
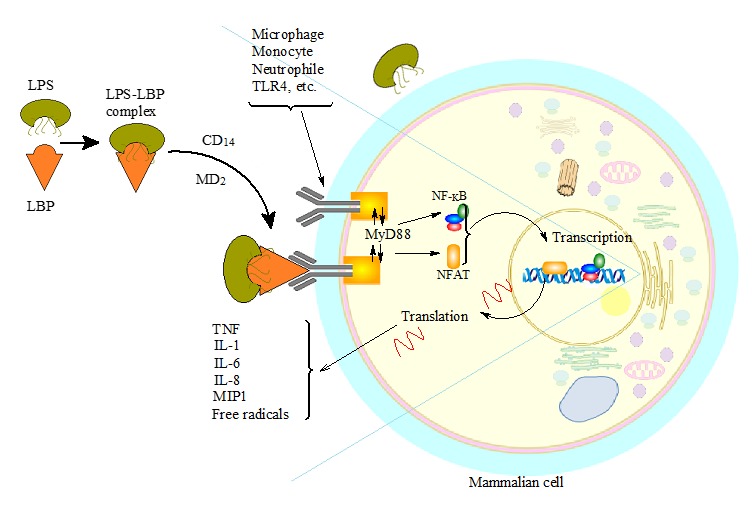
A typical endotoxin-protein interaction. LPS binds with LPB to form LPS-LPB complex, which is capable of interacting with TLR4 with the help of CD14 and MD2. The TLR4 interacts with MyD88 to trigger intracellular signals, which lead to the activation of cytokines to either clear infection (when LPS is in minute concentrations) or cause pathophysiological symptoms. LPS and endotoxin are considered synonymous (modified from: Raetz and Whitfield [22]).

In the microscopic perspective (*e.g.*, in the mammalian cell; **Fig. 2**), infused LPS binds and forms complexes with lipopolysaccharide-binding protein (LBP) and activates Toll-like receptor 4 (TLR4) [45]. The TLR4 is a member of the family of pathogen recognition receptors expressed by cells of the innate immune system (*e.g.*, monocytes, macrophages, neutrophils and dendritic cells). It recognizes structural motifs characteristic of PAMPs and its interaction with co-modulators, such as MD2 and CD14 [33,46,47]. MD2 and CD14 mediate the activation processes and allow TLR4 binding to LPS-LPB complex. The TLR4 possesses a large extracellular domain of leucine-rich units, a single transmembrane segment, and a smaller cytoplasmic signaling region that engages the myeloid differentiation primary response gene 88 (MyD88) adaptor protein [22,48,49]. The MyD88 adapter protein actuates transcription factors such as the nuclear factor of activated T-cells (NFAT) and the nuclear factor-κB (NF-κB) to spark the immune response. According to Beutler and Rietschel [45], NF-κB is the main transcription factor for the induction of the gene encoding mediators for cell sustenance. The activation process triggers the release of mediators, such as tumour necrosis factor α (TNF-α), interleukins (ILs), colony stimulating factor, prostaglandins, platelet-activating factor (PAF) and free radicals [8,45]. The NF-κB stimulates the production of proinflammatory cytokines (*e.g.*, macrophage inflammatory protein 1, MIP1) and type 1 interferons (IFNs) which are responsible for clearing infection or causing the pathophysiological effects of endotoxin exposure [8].

However, scientists are confident that the detrimental effects of endotoxicity could be controlled during upstream processing of biological products. We believe that thorough studies of the interactions and dynamics of endotoxin in a milieu of the biomolecular product of interest would contribute to an effective detection, quantification and removal remedies.

## LPS AND BIOMOLECULE INTERACTION

Gram-negative bacteria have been an important source of many useful bio-products, including proteins and biopharmaceuticals. This is owing to the relatively simple genetics and rapid growth rate of these beneficial bacteria coupled with their well-studied characteristics. These have resulted in their use in the commercial production of many life-saving products, *e.g.*, vaccines, insulin and other therapeutics. However, endotoxin—discussed as pyrogenic and yet an inherent part of Gram-negative bacteria—inevitably interacts, binds or forms complexes with the required isolated product, making the removal of endotoxin from microbial extracts a much needed though complicated task [8,50,51]. As a matter of emphasis, any thorough endotoxin removal strategy ought to be preceded by the analysis of its interaction with the biomolecule of interest. Such an exercise has a greater potential of discovering an effective removal strategy.

Remarkably, both the *in vivo* and *in vitro* endotoxin-biomolecule interactions have been investigated to some extent, resulting in the discovery of several techniques for endotoxin detection and separation from biomolecule. For instance, the interaction of LPS with lysozyme [52], plasmid DNA [53], green fluorescent protein (GFP) [54], antimicrobial proteins [55,56], and apolipoproteins [57] has been reported. Also, the identification of ionic interactions between LPS and biomolecules has played a central role in most removal strategies. This justifies that the thorough understanding of endotoxin-protein interaction is necessary for the development of any LPS removal strategy. In fact, some of these emerged endotoxin removal strategies are based not only on charge differences but also molecular mass differences. They include (but not limited to) anion exchange chromatography, cesium chloride density gradient centrifugation, Triton X-114 extraction, and polymyxin B chromatography [58]. These removal procedures are often elaborate, expensive and, more often, lacking general applicability [46]. An endotoxin removal strategy based on preferential binding of LPS to a stretch of cationic histidines, with the resulting complex capable of being trapped onto nickel affinity columns, has been presented [46]. However, there have been both qualitative and quantitative compromising issues with end-products, resulting from the available removal technique, hence the need to continually conduct research to overcome these limitations. A comprehensive review of existing endotoxin removal methods have been reported [7,59,60].

Among the number of biomolecules that show affinity/interaction with endotoxin are LBP, bactericidal/permeability-increasing protein (BPI), cationic protein, lysozyme, amyloid P component, lactoferrin and mammalian immune cells, such as monocytes and macrophages [8]. These interactions can be thought of as mostly driven by electrostatic forces, resulting from the diversity of the negatively charged groups of endotoxin and the possible positive charge of the proteins, which may as well be hydrophobic [53]. It has been postulated that the competition between proteins and endotoxins for Ca^2+^, in the form of protein-bound carboxylic groups and endotoxin-bound phosphoric acid groups, is liable to cause stable calcium linkages between them [53]. This exemplifies an endotoxin-biomolecule interaction which could be probed and modified as a separation technique.

**Figure 3.**
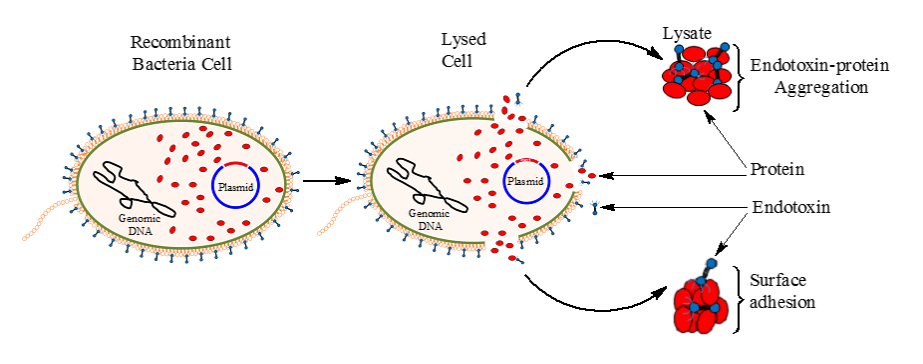
Endotoxin-biomolecule interaction. The firmly anchored endotoxin molecules get disengaged from the outer membrane of the cell during cell lysis for intracellular product extraction. They intercalate into the extract (biomolecule) and form stable aggregates. The endotoxin molecules also interact by surface adhesion. The recombinant protein is expressed by the insertion of interested gene into extrachromosomal DNA (plasmid). The endogenous plasmids have been omitted for clarity.

Generally, the injection of LPS into surrounding medium takes place during cell death, cell growth and cell division [3,18], shedding, proliferation or degradation [61], and even in the use of antibiotics [2,42]. For instance, the lysis of bacterial membranes when harvesting recombinant proteins from *E. coli* leads to the disengagement of endotoxin from the membranes and results in the contamination of the product [62]. The disengaged LPS associates with the protein medium upon release. The released LPS interact with the extracted biomolecule by surface adsorption, complexation/aggregation, or inclusions (**Fig. 3**) and, therefore, require studies to break linkages and separate the conjugates. Intriguingly, the investigation of biomolecular dynamics—in terms of complexation, affinity, localization and intermolecular distances—is one of the established strengths of FRET technique; thus, could provide insightful information and breakthrough in the quest to curb endotoxicity in terms of detection, quantification and subsequent removal strategies.

## ENDOTOXIN DETECTION AND QUANTIFICATION

Endotoxin detection is a necessary task for quality control in biological products, food and water safety, parenteral drugs, serological products, medical devices, recombinant and therapeutic products [2]. Moreover, protein products expressed in *E. coli* expression systems require intensive purification to remove endotoxin and reduce them to acceptable levels. However, the similarity of endotoxin to proteins in complexity, coupled with possible interference from added formulation components, renders endotoxin detection and quantification an essential yet challenging area of study [19]. The challenge in endotoxin detection and quantification, arising from the association of LPS with proteinaceous and other lipophilic substances, has driven the focus of many researchers towards the need to investigate a rapid and sensitive technique (*e.g.*, biosensors) for the detection and monitoring of endotoxin levels even in minute concentrations [2].

Currently, there are a lot of methods (**Table 1**) employed in detecting endotoxin. Many laudable efforts have been made to aid in the detection and quantification of endotoxins over the years. Several others are also under constant review and continual improvement. The first of the known techniques is the Rabbit Pyrogen Test, which was developed based on the endotoxin-induced pyrogenic response that is exhibited by a rabbit when infused with an endotoxin contaminated substance [63]. Its use was abolished, perhaps, due to claims by the animal right activist.

Another detection technique that is receiving constant improvement is the limulus amebocyte lysate (LAL) gel assay. Also known as the gel clot assay, the LAL gel method provides a simple qualitative, positive/negative result and is frequently cited in the majority of pharmacopeial monographs as the endorsed referee test [2]; that is in case discrepancies in endotoxin potency arise among different determination methods. The LAL gel is an *in vitro* assay based on the observation that lysate from horseshoe crab (Limulus polyphemus) amebocyte clots in the presence of very small levels of endotoxin of about 0.03 Endotoxin Units (EU)/ml [63].

The mechanism of the clotting process is well understood. Chen and Mozier [19] explained that endotoxin triggers a proteolytic cascade in the LAL, which leads to the cleavage of coagulation proteins (*e.g.*, Factor C) that coalesce and cause the reaction mixture to become turbid and form gel-clots. The degree of the turbidity putatively results from the magnitude of the endotoxin present.

The LAL gel test is believed to be sensitive to the presence of endotoxin; however, it significantly lacks robustness and repeatability, especially in the detection of material-bound LPS and other microbial contaminations [64]. It is strongly sensitive to disturbances in the form of pH, proteases, divalent cations, chelators, anticoagulants and serum [42]. Moreover, this assay is known to be laborious and liable to batch-to-batch variability [42]. Consequently, new and consistent endotoxin detection strategies are in high demand due to the limitations of the conventional LAL assay [42].

By way of surpassing limitations of the conventional LAL method, two kinetically monitored LAL-based endotoxin detection and quantification methods have been introduced, namely: chromogenic (kinetic chromogenic and endpoint chromogenic) and turbidimetric techniques. However, a test conducted by Chen and Mozier [19] to compare the different LAL methods for endotoxin detection in protein milieu suggested that one should consider the expression system, formulation and potential sources of contamination when selecting LAL assay. The above suggestion stems from significant data variances noted in some samples as recorded in Chen and Mozier [19].

In parallel with Chen and Mozier [19], Das *et al*. [2] explained explicitly that the endpoint chromogenic LAL (QCL-1000) is a quantitative test and offers less product interference; the kinetic chromogenic LAL (KQCL) provides greater sensitivity with minimized product interference for proteins, vaccines, and other biologicals; and the kinetic turbidimetric LAL (Turb) offers a cost-effective choice for water and parenteral fluids. In consequence of these drawbacks, there is a nascent search for a superior technique, which is devoid of these conditions, to ease up complications associated with endotoxin detection and quantification. Nevertheless, consistent results could only be achieved if the mechanisms of the interactions were well-understood and characterized at both micro and macromolecular levels, making FRET studies an option to consider.

Most recently, a recombinant Factor C (rFC) method, named PyroGene, was introduced to the market, and it is believed to be sensitive to only endotoxin [19]. Nevertheless, most of the LAL methods have complications with glucan structures and, to some extent, lipids; thus, posing a challenge regarding method selection and development. In response to this challenge, Kim *et al*. [11] developed an electrochemical aptasensor with significantly improved detection time contrary to the conventional LAL assay. The aptasensor showed negligible cross-binding reactivity to various biomolecules — such as pDNA, RNA, proteins, saccharides, and lipids — which frequently coexist with LPS in many bioprocessing liquors. There are several methods in the literature of the past few years suggesting that the future of these assays could depend on biosensors. For instance, protein-based [65-69], peptide-based [70-74], antibody-based [75-78], aptamer-based [11,79-83], cell-based [64,84-87], and other material-based [88-91] biosensors and assays have emerged. The notable trend of emerging LPS detection techniques is in association with the quest for simplicity and stability [92,93]; sensitivity and ease of handling [20]; reproducibility and reliability [94]; and, finally, robustness and cost efficiency [42].

Based on the challenges in current endotoxin detection techniques, as outlined in **Table 1**, there is clearly the need to modify existing methods or develop new approaches that are superior to the conventional methods. Moreover, the separation and purification techniques employed in the treatment of endotoxin contaminated bioproducts demand a significant level of enhancement. Interestingly, most biological phenomena dwell on the fundamental physiological and biochemical processes consisting of molecular binding and association, conformational changes, molecular diffusion, and catalysis [95]. The elicitation of these biological phenomena is dependent on the understanding of the spatio-temporal distributions and functional states of the constituent molecules [95]. Apparently, FRET techniques offer such platforms and could be the next frontier for endotoxin elucidation.

## FÖRSTER RESONANCE ENERGY TRANSFER

FRET is an important technique for studying the different aspect of interactions between biomolecules in their natural environments [96]. It allows the investigation of molecular processes in nanometer resolution [96]. Such resolutions are appropriate for most biomolecules or their constituent domains undergoing complex formation and conformational transition [95]. The tool provides a way of measuring and understanding different biological systems and molecular interactions [97]. FRET has been known to be well-suited for investigating protein-protein and protein-ligand interactions within close proximity, *i.e.*, 1–10 nm [98,99] and could as well be applicable in endotoxin-protein investigations.

The application of FRET has exploded over the years. This has been partly attributed to the ability of FRET to combine with advanced techniques such as fluorescence lifetime imaging (FLIM), fluorescence correlation spectroscopy (FCS), fluorescence cross-correlation spectroscopy (FCCS) and fluorescence recovery after photo-bleaching (FRAP). These FRET complementation are capable of providing excellent resolution of molecular images at distances beyond the diffraction-limited spatial resolution of optical microscopy [98,100]. Obeng *et al*. [101] has comprehensively reviewed the salient applications and prospects of FRET over the years.

## FUNDAMENTAL PRINCIPLES OF FRET

The core principle of FRET involves the transfer of excitation energy from a donor fluorophore to a nearby acceptor fluorophore (or chromophore) in a non-radiative fashion *via* long-range dipole-dipole interaction [102-104]. The fluorophore pairs are molecular dipoles with intrinsic ability to fluoresce upon laser excitation and, thus, have been a means to report biological occurrences. These fluorophore pairs are mostly fluorescent proteins (*e.g.*, GFP and GFP-like proteins such as cyan, yellow, orange, and red fluorescent proteins), synthesized non-protein organic fluorophores/dyes (*e.g.*, cyanine dyes, Alexa-Fluo dyes, fluorescein, rhodamine, *etc*.) and luminescent nanocrystals (quantum dots), with the last two classes currently opening vistas in FRET-based experiments. Notably, the resonance frequencies of fluorophore pairs are classically similar and the donor transfers energy at the same frequency at which the acceptor absorbs photons. The individual fluorophores are conjugated to different biomolecules of interest to facilitate the investigation of interactions between the biomolecules.

**Figure 4.**
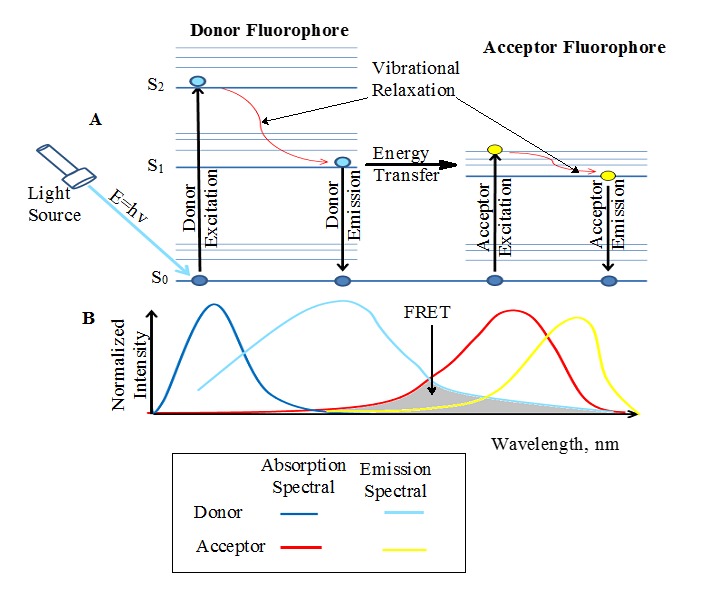
The resonance energy transfer principle. **A.** Jablonski diagram; **B.** Spectral image. The donor fluorophore absorbs energy from the incident light, which causes it to move from the ground state (S_0_) to higher energy states (S_1_ and S_2_). Upon close contact (1–10 nm) with the acceptor fluorophore, the donor transfers excitation energy non-radiatively to the acceptor to cause it to fluoresce. There is no emission of photons at the point of energy transfer. When all conditions are met, the donor emission spectrum overlaps the acceptor absorption spectrum to signify the occurrence of FRET.

During FRET, the donor fluorophore acquires excitation energy from an incident beam of light, which takes it from a lower energy (ground) state to an excited state thereby displaying fluorescence (**Fig. 4**). However, in the presence of an appropriate acceptor fluorophore (quencher), the donor non-radiatively couples some of its excitation energy to the acceptor when they are 1–10 nm apart within the electromagnetic field. This transferred energy excites the electrons of the acceptor, leading to a decrease (quenching) of the donor radiance and fluorescence lifetime. The remaining energy is putatively dissipated in the form of heat and vibrational relaxation, thus, complying with the law of conservation of energy. It is worth noting that the resonance energy transfer occurs only within the electromagnetic field created by the excited donor, and it rapidly decays over distance [105].

Although there is a proposed transfer of electromagnetic energy from the donor to acceptor, there is no emission of photons in the process; rather, the electromagnetic wave is modulated through the fluorescent dipoles in the same way as a radio antenna [99]. Therefore, through ‘resonance coupling’ between the chromophores, the donor radiance decreases or quenches, resulting in a reduction of excited state lifetime, whereas the acceptor fluorescence increases as the energy transfer transpires [103]. The complex quantum electrodynamic theories of FRET is beyond the scope of this review; however, technical details of FRET have been comprehensively published in the literature [103,106-109].

The FRET principle helps in the elucidation of the interaction between two molecules; the interaction is mostly predicted based on the efficiency of the resonance energy transfer. According to the Förster theory [104] and its derivations, the efficiency of FRET (E_FRET_) is related to the intermolecular distance (r) between the donor (D) and acceptor (A) molecules as follows:
(Eqn. 1)
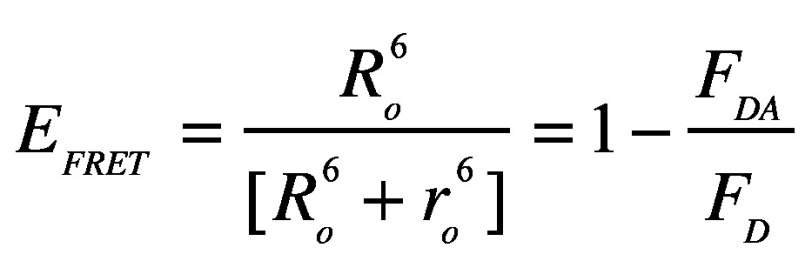

(Eqn. 2)
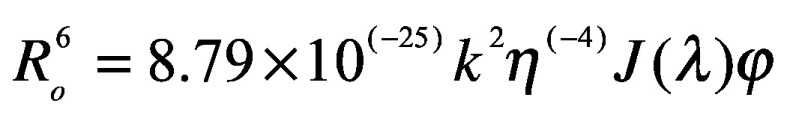


The E_FRET_ correspondingly relates with the fluorescence intensity of donor in the presence (*F*_DA_) and absence (*F*_D_) of the acceptor molecule [110], and it represents the ratio of transferred ‘resonance’ energy to the total photonic energy absorbed by the donor [111]. The *R*_O_ is the characteristic distance whereby the E_FRET_ is 50%, and it can be estimated for any pair of fluorophores. The *R*_O_ is associated with the relative alignment or orientation (*k*^2^), the refractive index of the medium (*η*), the quantum yield of the donor (*Φ*), and the extent of overlap (*J*(*λ*))between donor emission and acceptor absorption spectra. It is different for different fluorophore pairs. For most useful FRET pairs, the value of falls between 3 and 8 nm; for example, the magnitude of *R*_O_ for the most common donor-acceptor FRET pairs, cyan fluorescent protein (CFP) and yellow fluorescent protein (YFP), is 4.92 nm [105,112].

FRET is a condition bound phenomenon and, therefore, without the fulfillment of the requirement for FRET, there will be no ‘fretting’ of molecules. These prerequisites for FRET are: (1) the separation distance between the donor and acceptor must be in close proximity, characteristically in the range of 1–10 nm; (2) the emission spectrum of the donor chromophore must overlap the absorption (excitation) spectrum of the acceptor; (3) the relative alignment of the donor-acceptor transition dipole moments must be adequate; and (4) the fluorescence lifetime of the donor molecule must be of sufficient duration. A limitation in any of these conditions would mean ‘no-FRET’ occurrence.

## PROMISING CONCEPT OF FRET

The promising application of FRET in biomolecular research began in the early 1970s but was limited regarding conjugating chromophores to biological samples. However, with the rapid advancement of molecular biotechnology, the use of FRET has increased tremendously over the past few years [99,105]. The surge in the application of FRET in biology related research came up shortly after the engineering of GFP. Presently, GFP-based FRET has provided researchers with a very powerful tool to investigate molecular interactions in living cells (in the range of seconds) and follow such *in vivo* interactions in a non-invasive manner [105,113].

Over the years, FRET has found numerous techniques and diverse applications in both intramolecular and intermolecular studies of biomolecules. For example, intramolecular FRET between fluorescent proteins (FPs) attached to opposite ends of responsive peptide or protein has ensued in the development of many integrated biosensors, with a vast majority yielding positive results [99].These biosensors, through the application of fluorescent indicators, are capable of use in the recognition of specific target analytes and even a group of closely related analytes in test matrix [2], and may not be limited to endotoxin contaminated media.

Intermolecularly, FRET has been used in the investigation of the structure [114], conformational changes [115], biomolecule interactions [98,116-118], and as a powerful spectacle of biochemical events [119,120]. The technique has also found significant function in membrane fusion assays and real-time polymerase chain reaction (PCR) assays; thus, becoming a standard method for detection and quantification in many applications, including pathogen detection and expression analysis [121].

Moreover, FRET has proven to be one of the most powerful methods for hetero-oligomerization investigation. For instance, it allows diverse approaches to investigate molecular binding characteristics, such as the determination of the ligand-protein binding characteristics, the number and position of binding sites, and the detection of binding-induced conformational transitions [115,122]. It is worth mentioning that the application of FRET for studying specific antibody-antigen [123,124] and protein-drug [125,126] interactions in biotechnological and pharmacological studies are not far-fetched, and some of these approaches employed are transferrable, especially for endotoxin-biomolecule interaction analysis. It stands to reason that these practices solicit to be mimicked in the investigation of endotoxin and its characterization to deepen the conceptual understanding of its activities in the presence of biomolecules and to help mitigate the prevailing challenges.

Interestingly, the technique has been identified as cost efficient and exquisitely sensitive for the monitoring of molecular interactions and conformational changes *in vivo* [58]. Moreover, it requires little materials and relatively inexpensive instrumentation [122]. However, it is disappointing that the prospects of using FRET in endotoxin-biomolecule interaction investigations have been ill-explored, though results obtained from such studies could help solve current endotoxin detection and quantification drawbacks. The few associated reports in the literature are mostly based on fluorescence-mediated biosensing.

For instance, Jones and Jiang [127] used fluorescence spectroscopy based on absorption spectra shift and ratiometry to study LPS and its interaction with spermine-pyrene (Sp-Py) conjugates in aqueous solution. Their work reported the aggregation of the amphiphilic fluorescent Sp-Py conjugate in the presence of the equally amphiphilic LPS. A similar attempt was replicated by Zeng *et al*. [128] but was based on the supramolecular assembly of pyrenyl quaternary ammonium. Also, Lim *et al*. [129] employed the technique of fluorescence recovery to develop peptide-assembled graphene oxide as a sensor for detecting endotoxin.

Interestingly, ratiometric FRET and other FRET techniques are equally capable of yielding the same results and even giving extra details such as the binding dynamics, conformational specificities, localization and the intermolecular distances between the LPS and the relevant molecule. These spatial and temporal details obtainable *via* FRET studies are pertinent for the establishment of a superior LPS detection, quantification and removal strategies. Regarding the fabrication of FRET-based biosensor for LPS detection, the approach would involve the separate tagging of endotoxin and the “affinity” molecules with donor and acceptor fluorophores, respectively. The next step would be to measure FRET signals of (1) the donor, (2) the acceptor and (3) their interaction in both blank and analyte media (*e.g.*, processing fluids). Finally, the biosensor response would be an algorithmic superimposition of all the signals to account for qualitative (presence [+] or absence [−]) and quantitative demands [101].

Correspondingly, Voss *et al*. [130] demonstrated the use of FRET to study the interaction of LPS and some amphipathic lipid with the CD14-derived peptide in a quest to fabricate a sensor to detect the presence of LPS. Their recommendable approach, which capitalized on the affinity of LPS to bind to CD14, resulted in a highly specific sensor of about three orders of magnitude in specificity above synthetic LPS sensors [130]. In spite of this achievement, data on the interaction of the LPS and essential target biomolecules (*e.g.*, proteins, vaccine, therapeutic drugs, *etc*.) even in the presence of cell-lysing chemicals and extraction sorbents are woefully deficient in the literature. Most of the endotoxin removal strategies currently existing employ the use of detergents, chelating agents, chaotropic agents, or bile salt. However, the underlying mechanism behind these procedures is less understood; thus, explaining the incessant challenge. Fortunately, such an interaction could be highly captured under the nanometer resolution of FRET with appreciable insights.

It is evident, from the scope of published literature on endotoxin investigation, that the understanding of endotoxin-biomolecule interplay based on high-resolution imaging techniques is suffering a dearth. Although some researchers have demonstrated the use of Fourier transform infrared (FTIR) microscopy [87], UV/visible absorption and steady-state fluorescence spectroscopy [127,131] and electron microscopy [57] in the investigation of endotoxin and biomolecules, it is obvious that much more could be achieved with FRET. It is worth emphasizing that the challenge of current biochemical industry, apropos endotoxin contamination in the production of recombinant injectants and other essential biochemicals, necessitates the acquisition of more information about the interaction of LPS and these vital bioproducts. In addressing this, LPS can be reengineered in Gram-negative bacterial expression system to possess an FP-tag, such that the *in vivo* interaction with the relevant biomolecule (also with a tag) becomes traceable. In a similar fashion, fluorescent dyes could be used for *in vitro* investigation. The ensuing information is what will be of relevance in the planning of better ways of detecting, quantifying and reducing endotoxin contamination in biomanufacturing. The preliminary information regarding the unique behavior of endotoxin at different manufacturing conditions (temperature, concentration, pH, type of salt and type of byproducts) could help engineers develop a process that best minimizes endotoxin-bioproducts interaction hence the ease of product purification.

**Figure 5.**
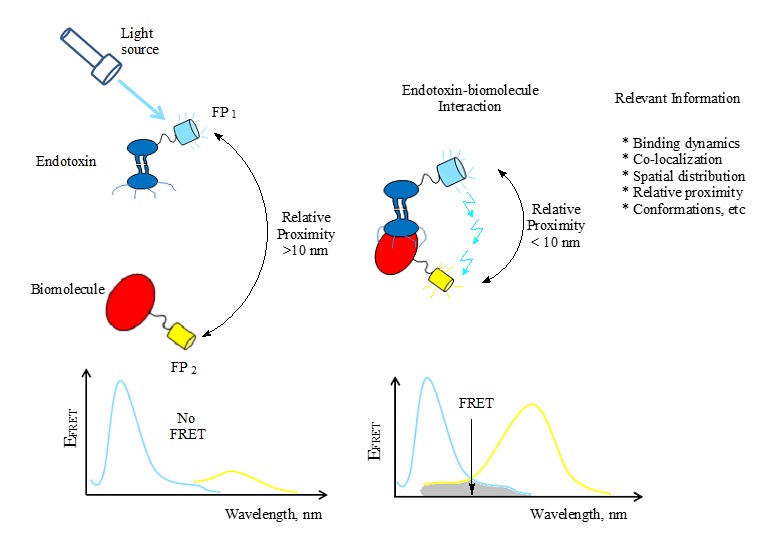
FRET-mediated endotoxin-biomolecule interaction. The interaction of endotoxin-FP_1_ and biomolecule-FP_2_ during FRET reveals insightful information relevant for endotoxin detection, quantification and subsequent separation in protein milieu. There will be no FRET if the associated conditions are not met.

In principle, the application of FRET in endotoxin-protein interaction analysis (**Fig. 5**) may not be much different from that of the most practiced protein-protein interaction analysis. Moreover, the fundamental conditions that need to be satisfied before FRET occurs will remain unchanged since FRET is a condition bound mechanism. However, the application of FRET in endotoxin-biomolecule investigations may, perhaps, necessitate simple algorithmic modifications based on the fundamental principles of FRET; thus, an area requiring investigation.

## THE BIG CLUE

There is plausibly a high possibility of using FRET analysis to investigate and extend the frontier of current knowledge on endotoxin-biomolecule interaction. With the application of FRET, scientist could commonly investigate/monitor: (1) the binding kinetics between molecules of endotoxin and the biomolecule of interest; (2) the localization and spatial distribution of endotoxins and biomolecules in complexes; (3) the relative proximity of the molecules; (4) the endotoxin-biomolecule physiological responses and conformational changes; and (5) the endotoxin-biomolecule high-resolution FRET images.

These outlines are generally the stronghold of FRET; however, the existing body of literature is in paucity of such information. Cho *et al*. [100] has presented a simple but robust FRET method which is putatively applicable in the above mentioned. The outcome of such investigations could help develop FRET as a sensitive identification and quantification tool for endotoxin-biomolecule assessment. Moreover, FRET-mediated elucidations of endotoxin-biomolecule affinity, aggregation, and associated binding dynamics could be relevant for the development of separation procedures and anti-binding/aggregation strategies during bioprocessing. For instance, since molecular binding is a reversible equilibrium process governed by the law of mass action, process solvents which competitively retard molecular binding and other associations could be explored from binding kinetics data obtainable *via* FRET [132]. Such information could help in the modification or tuning of chromatographic (monolithic) stationary phases to selectively trap endotoxin to obtain highly purified products. This achievable reinforcement in endotoxin removal methods would significantly resolve endotoxin contamination in bioprocessing. This clarion call then becomes a multidisciplinary task, requiring the expertise of scientists such as spectroscopists, biophysicists, and biotechnologists.

## SOME POSSIBLE FRET CHALLENGES

The main challenge in FRET studies is in association with materials and instrumentation selection. In terms of the materials, one possible challenge would be in association with the stability differences between the endotoxin and the biomolecule of interest. The thermal and chemical stabilities of endotoxin are significantly superior to that of most biomolecules [7,8,46] therefore, pH and temperature of their milieu should be carefully selected in order not to denaturize the biomolecule during the investigation. Also, the selection of workable fluorophore pairs suitable for endotoxin-biomolecule FRET investigations should not be compromised. Sometimes the large size of FP pairs may reduce accessible interaction space and cause inflated FRET efficiency values. However, a good choice of fluorophore pairs prevents or subdues FRET related issues such as the occurrence of “unplanned” homo-fretting (which normally occurs between identical fluorophores), spectral cross-talk (or spectral bleed-through), autofluorescence and other quantum yield determination challenges. The authors have provided an extended discussion on FRET-associated challenges covering instrumentation and other technicalities in their recent publication for the interest of readers [101]. The authors further recommend some important literature [99,108,109] that have addressed FRET, its associated challenges and some experimented remedies.

## CONCLUSION

The deficiencies in the existing knowledge on endotoxin-biomolecule interactions are significant contributors to the persistent challenges in downstream separation and purification of cell culture-based bio-products, especially regarding Gram-negative bacteria. FRET spectroscopy could complement and consolidate existing knowledge on endotoxins and biomolecules, as well as their interplay. Such results are prerequisite for the development of efficient biomolecule purification strategies. The promising advantages of FRET should echo resonating ideas in the minds of researchers to explore its possible use for endotoxin-biomolecule studies and subsequent development of endotoxin identification and quantification tool.

## Figures and Tables

**Table 1.** Some LPS detection methods and challenges associated^a^.

LPS detection methods	Sub-techniques	Detection limit (as low as)	Challenges/Defects
Rabbit pyrogen test		0.5 EU^b^/ml	Limited sensitivity Less accurate Large samples require large number of animal models Cost intensive Time consuming
Limulus amebocyte lysate assay	Gel-clot^c^ Chromogenic Turbidimetric Viscometric	0.03 EU/ml	Limited specificity to target Lacks repeatability and robustness Free metal ions inhibition Susceptible to proteases Reactivity with polymeric forms of glucose (*e.g.*, glucan)
Monocyte activation test		10 pg/ml	Source^d^ inconsistency Limited availability of source Less stable over a large number of assays
Bovine whole blood assay		0.04 EU/ml	Source^e^ acquisition intricacies Whole blood inconsistencies Less stable over a large number of assays
Biosensors	Protein-based Peptide base Antibody-based Aptamer-based Cell-based	100 pg/ml 0.2 ng/ml 20 pg/ml 8.7 fg/ml 0.5 μg/mL	Limited specificity to target Limited sensitivity Unwanted LPS adsorption Loss of target molecules Significant error margins Cost intensive Complex detection process Time consuming Labor intensive Complex fabrication procedures Complex biological reactions

^a^The content of **Table 1** was gathered from [20], [42], [64], [57] and [133].

^b^EU equals approximately 0.1–0.2 ng endotoxin/ml [86].

^c^Traditional method.

^d^Human blood.

^e^Bovine blood.
